# Performance of HbA1c versus oral glucose tolerance test (OGTT) as a screening tool to diagnose dysglycemic status in high-risk Thai patients

**DOI:** 10.1186/s12902-019-0339-6

**Published:** 2019-02-15

**Authors:** Yotsapon Thewjitcharoen, Amia Jones Elizabeth, Siriwan Butadej, Soontaree Nakasatien, Phawinpon Chotwanvirat, Ekgaluck Wanothayaroj, Sirinate Krittiyawong, Tinapa Himathongkam, Thep Himathongkam

**Affiliations:** Diabetes and Thyroid Center, Theptarin Hospital, Bangkok, Thailand

**Keywords:** HbA1c, OGTT, Dysglycemia, Performance, Diagnostic accuracy

## Abstract

**Background:**

Dysglycemic status defined by prediabetes and diabetes is known to be related with future risk of diabetic complications and cardiovascular diseases. Herein, we aimed to determine the diagnostic accuracy of glycated hemoglobin (HbA1c) when compared with oral glucose tolerance test (OGTT) as a reference test in identifying dysglycemic status among high-risk Thai patients receiving care in an out-patient setting.

**Methods:**

An 11-year retrospective cross-sectional study of high-risk Thai patients who underwent OGTT during 2007–2017 was analysed. The OGTT was used as a reference test to identify subjects of dysglycemic status. The diagnostic accuracy of HbA1c and the agreement between HbA1c and OGTT were examined. Validated Thai diabetes risk score, Thai cardiovascular risk score (Thai CV risk score), and visceral fat area (VFA) were also compared in each glycemic status from OGTT as surrogate markers for future diabetes and cardiovascular diseases.

**Results:**

A total of 512 subjects (females 60.5%, mean age of 50.3 ± 12.7 years, BMI of 26.5 ± 4.6 kg/m^2^) were reviewed. Normal glucose tolerance (NGT) was found in 220 patients (43.0%), impaired glucose tolerance (IGT) in 191 patients (37.3%), and diabetes in 101 patients (19.7%). The prevalence of diabetes using OGTT was approximately two times higher than those defined by HbA1c (19.7% versus 11.1%). There were poor agreements between the classifications of prediabetes and diabetes defined by OGTT and HbA1c (Cohen’s Kappa 0.154 and 0.306, respectively). Using a cut-off value for HbA1c ≥6.5% as a threshold for HbA1c-defined criteria of diabetes, sensitivity was 32% (95% CI 23–41%) and specificity was 94% (95% CI 92–96%). The optimal cut-off HbA1c value for detecting diabetes by Youden’s index was at HbA1c 6.2%. Thai CV risk score was much higher among the OGTT-defined diabetes group when compared with the NGT group (median score 10 vs. 3, *p*-value < 0.001).

**Conclusions:**

Despite the practicality and validity of HbA1c as a diagnostic test, our study suggested that HbA1c as a screening tool for diabetes in high-risk Thai patients is much inferior to OGTT. With limitations of HbA1c, physicians should continue to advocate OGTT as a screening tool for the identification of dysglycemic status in high-risk Thai patients.

## Background

Approximately 451 million (8.8%) adults worldwide are expected to have diabetes, and the number is estimated to reach 693 million (9.9%) by the year 2045 [1]. In the Western Pacific Region, including Thailand, the prevalence of diabetes is estimated to increase up to 15% in 2045 [2]. Dysglycemia, both diabetes and prediabetes, has been defined using diabetic retinopathy as a specific complication of diabetes and these hyperglycemic states are associated with cardiovascular diseases and metabolic syndromes [3]. Prediabetes includes individuals with impaired fasting glucose (IFG) or impaired glucose tolerance (IGT) or elevated glycated hemoglobin (HbA1c) [4]. According to previous studies [5, 6], up to 70% of people with prediabetes will eventually develop overt diabetes during their lifetime. The annual incidence of progression from prediabetes to diabetes is around 5–10% depending on the population characteristics and the definition of prediabetes (6–9% in subjects with isolated IFG, 4–6% in those with isolated IGT, up to 15–19% among those with both IFG and IGT, and subjects with HbA1c levels from 5.7–6.4% have a 7.5-year predicted risk of 43.1% for incident of diabetes). Moreover, individuals with prediabetes are at an increased risk of cardiovascular diseases (CVD) and premature mortality when compared with subjects with normoglycemia [7].

Currently, there are three glucose-based diagnostic methods with specific cut-off points for diagnosing dysglycemic status [3, 8]. HbA1c is the latest method and the most convenient screening tool for dysglycemia, but it is also known to be less sensitive than the oral glucose tolerance test (OGTT). It is still debatable whether HbA1c or OGTT should be the preferred test for diagnosing diabetes [9, 10]. The results from the Detection Strategies for Type 2 Diabetes and Impaired Glucose Tolerance (DETECT-2) study [11] which included more than 40,000 participants with gradable retinal photographs from five countries did not support the superiority of OGTT over HbA1c or fasting plasma glucose (FPG). Also, the effect of race/ethnicity on HbA1c level were apparent [12, 13]. A previous study from a community-based diabetes prevention program in high-risk Thai participants [14] revealed that 51% of total participants were positive for dysglycemia (defined by either FPG or OGTT) and the prevalence of diabetes classified by OGTT was two times higher than those defined by FPG (11.0% versus 5.4%). Unfortunately, no study was done to evaluate the clinical utility of HbA1c compared with OGTT to diagnose dysglycemia in Thai participants. The objectives of this study were to examine the diagnostic accuracy of HbA1c using OGTT as a reference standard to identify subjects of dysglycemic status and also evaluate the agreement between HbA1c and OGTT in diagnosing dysglycemic status among high-risk Thai patients.

## Methods

We reviewed a sample of high-risk adult Thai patients, aged 15 and older, who underwent 75-g OGTT and had an HbA1c value within 3 months of OGTT procedure during the study period of 2007–2017 at Theptarin Hospital, Bangkok, Thailand. Most subjects underwent OGTT due to high risk for diabetes such as having a body mass index (BMI) ≥25 kg/m^2^ or having abdominal obesity, Thai diabetes risk score ≥ 6, have a history of IFG, or a family history of diabetes. Thai diabetes risk score is a validated risk score calculated using factors including age, sex, BMI, waist circumference (WC), hypertension, and family history of diabetes for predicting diabetes over 12 years in Thai people [15]. Subjects were excluded from this study if they had a history of diagnosed diabetes, had hematologic or endocrinologic disorders or on medications that would interfere with glucose metabolism. Subjects that are pregnant during the OGTT and subjects with other nationalities were also excluded. Only the results of the first OGTT in the study period were used for the analysis.

Data were collected on baseline characteristics such as, age, sex, blood pressure, BMI, WC, history of diabetes in first-degree relatives, previously documented cardiovascular diseases, history of smoking, hypertension, plasma lipid profiles, and statin usage. Visceral fat area (VFA) calculated using body composition analysis within 1 year of OGTT was retrieved. A Thai cardiovascular risk score (Thai CV risk score) developed from Electricity Generating Authority of Thailand **(**EGAT**)** study [16] was also calculated to evaluate the risk in individual for future cardiovascular diseases. Thai CV risk score aimed to quantify the estimated 10-year absolute cardiovascular risk for each individual. Variables used for calculating include age, sex, smoking status, diabetes status, WC, height, systolic blood pressure, and/or total cholesterol, HDL, LDL [17]. Standards for Reporting of Diagnostic Accuracy Studies (STARD) 2015 checklist was followed with HbA1c as the index test and OGTT as a reference standard to ensure the proper methods for studying diagnostic accuracy test [18]. This study was reviewed and approved by the Theptarin Hospital ethics committee (EC No.07/2017).

### Definition of prediabetes and diabetes

In this study, the definition is based on 2018 American Diabetes Association (ADA) criteria [3]. Diabetes was defined as subjects with 2-h plasma glucose from OGTT ≥200 mg/dL and/or HbA1c ≥ 6.5% and/or FPG of ≥126 mg/dL. The term “prediabetes” refers to IFG (FPG 100–125 mg/dL), IGT (2-h plasma glucose from OGTT at 140–199 mg/dL) or an HbA1c level of 5.7–6.4%. Normal glucose tolerance (NGT) was defined as subjects who had 2-h plasma glucose less than 140 mg/dL.

### Laboratory investigation

After fasting for at least 8–12 h, OGTT was performed 2 h after the ingestion of a standard 75-g glucose load. Plasma glucose was measured by enzymatic hexokinase method (Roche Diagnostics Cobas analyzer). Measurement of HbA1c was done by electrochemiluminescence immunoassay using Abbott Diagnostics core laboratory (from 2007 to 2008) and Roche Diagnostics Cobas Analyzer (from 2009 to 2017). The HbA1c test was DCCT-aligned assay and was accredited by the National Glycohemoglobin Standardization Program (NGSP).

### Statistical analysis

Continuous variables were presented as mean (±standard deviation, SD) or median (interquartile range), and categorical variables were presented as proportions. Subjects were divided into 3 groups NGT, IGT, and DM according to OGTT criteria. Clinical characteristics were compared using one-way ANOVA and post hoc analysis. *P-value* ≤ 0.05 was considered statistically significant. The diagnostic accuracy of HbA1c to diagnose diabetes when OGTT was used as the reference standard was expressed in four dimensions (sensitivity, specificity, positive likelihood ratio and negative likelihood ratio). The Youden’s index which combines sensitivity and specificity into a single measure (sensitivity + specificity - 1) was calculated at selected HbA1c thresholds to define diabetes from OGTT. The agreement between HbA1c and OGTT was analyzed using Cohen’s kappa coefficients (K). The receiver operating characteristic (ROC) curves, plotted by using sensitivity and 1-specificity, were used to calculate the performance of the HbA1c as area under curves (AUC) in diagnosing prediabetes and diabetes using the OGTT as the reference. All statistical analyses were conducted using the Statistical Package for the Social Sciences (version 22.0; SPSS, Chicago, IL, USA).

## Results

### Demographic and clinical characteristics

**Fig. 1:**
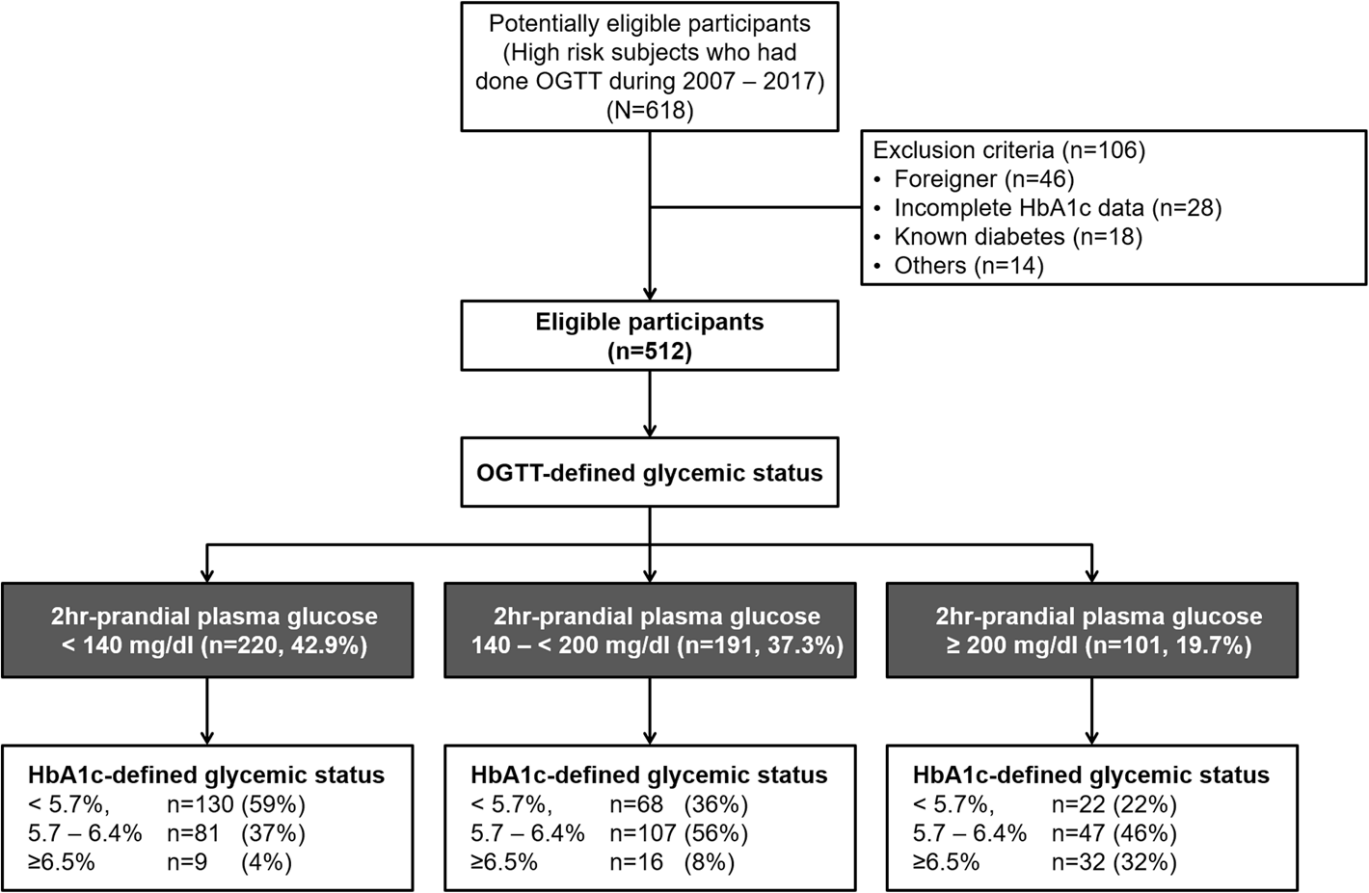
Flow diagram of the study according to Standards for Reporting of Diagnostic Accuracy Studies (STARD)

**Fig. 2:**
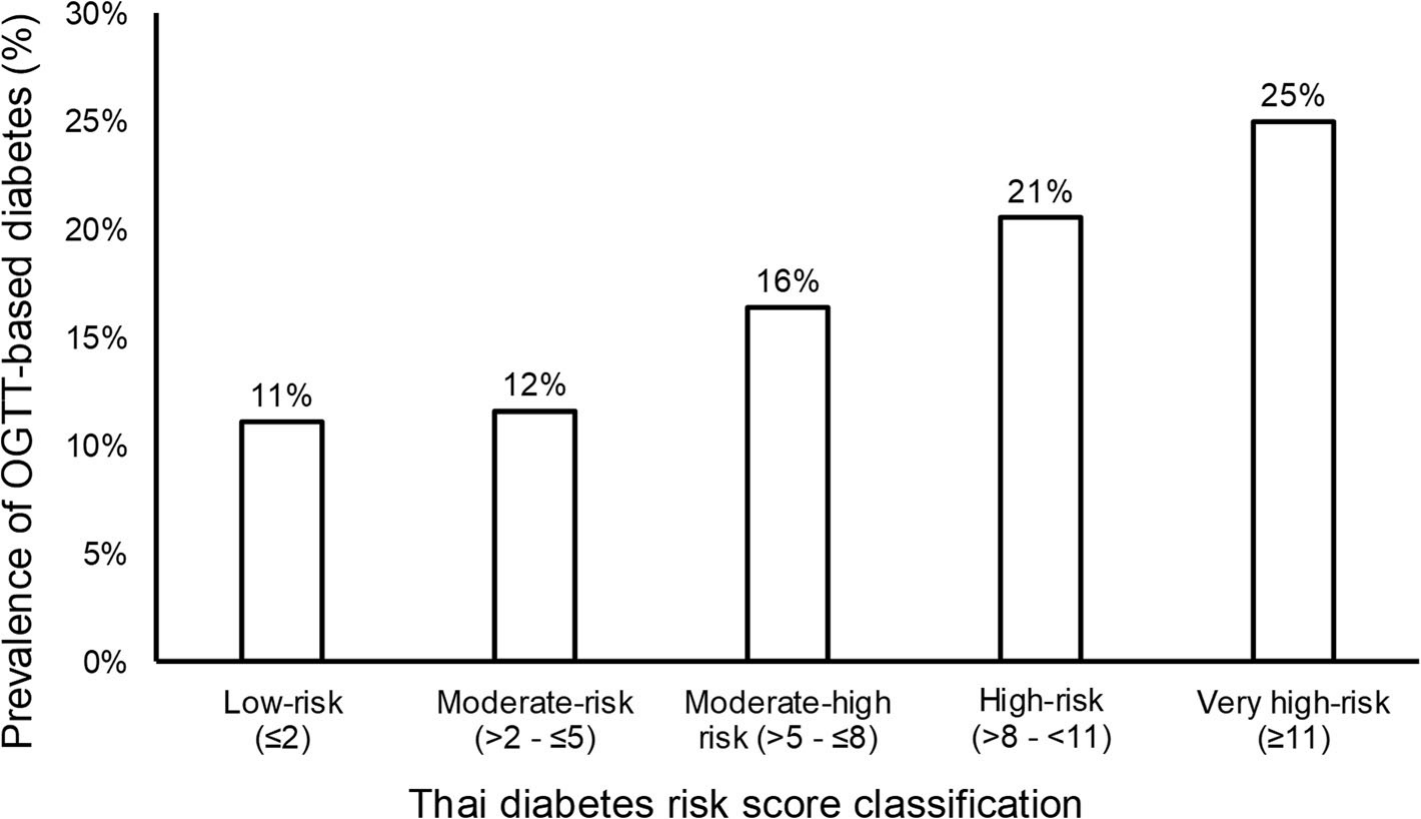
The distribution of OGTT-based prevalence of DM based on Thai diabetes risk score

Of the 618 subjects without prior history of diabetes who underwent OGTT from 2007 to 2017, 512 subjects met the inclusion criteria for analysis as shown in Fig. 1. Of these subjects, 74.8% had Thai diabetes risk score ≥ 6 out of the total score of 17, 62.9% had a history of IFG prior to the study, 59.4% had a BMI ≥ 23 kg/m^2^. According to the results of OGTT, dysglycemia was found in almost 60% of the subjects (37.3% found IGT and 19.7% was diagnosed with diabetes). Table 1 shows the clinical characteristics and laboratory data of the 512 participants that are categorized into 3 groups including NGT, IGT, and DM. The mean age of all subjects was 50.3 ± 12.7 years and the mean BMI was 26.5 ± 4.6 kg/m^2^. Compared to the NGT group, the IGT and DM groups were significantly older (*p* = 0.001) and tended to have higher BMI but did not reach statistical significance (*p* = 0.189). As shown in Table 1, there were significant differences in HbA1c in each glycemic spectrum (HbA1c 5.6 ± 0.4% in NGT, 5.8 ± 0.5% in IGT, 6.1 ± 0.5% in DM, *p*-value < 0.001). Moreover, Thai CV risk score was much higher in DM group when compared with NGT group (median score 3 vs. 10, *p*-value < 0.001). Based on Thai diabetes risk score, the distribution of OGTT-based DM prevalence varied from 11% in low-risk category (score ≤ 2) to 25% in very high-risk category (score ≥ 11) as shown in Fig. 2.

**Table 1:** Clinical characteristics and laboratory data of analyzed subjects classified by OGTT (*N* = 512)

	Overall (*n* = 512)	NGT (*n* = 220, 42.9%)	IGT (*n* = 191, 37.3%)	DM (*n* = 101, 19.7%)	*p*-value
Female (%)	60.5%	58.6%	58.6%	68.3%	0.205
Age (years)	50.3 ± 12.7	48.4 ± 12.8	50.3 ± 11.4	54.3 ± 14	0.001
BMI (kg/m^2^)	26.5 ± 4.6	26.1 ± 4.7	26.7 ± 4.5	27.0 ± 4.6	0.189
Systolic BP (mmHg)	126 ± 16	124 ± 16	126 ± 15	131 ± 15	< 0.001
Diastolic BP(mmHg)	74 ± 11	74 ± 11	75 ± 11	74 ± 11	0.578
Fasting plasma glucose	103 ± 12	98 ± 9	103 ± 11	112 ± 15	< 0.001
2-h plasma glucose	155 ± 51	111 ± 19	163 ± 17	236 ± 29	< 0.001
Hypertension	31.8%	27.7%	32.5%	39.6%	0.103
History of smoking	10.4%	11.0%	11.1%	7.9%	0.422
Statin usage	37%	34%	40%	40%	0.370
History of CVD	2.3%	1.8%	2.1%	4.0%	0.339
Family History of DM	60.2%	57.7%	59.7%	66.3%	0.339
HbA1c (%NGSP)	5.8 ± 0.5	5.6 ± 0.4	5.8 ± 0.5	6.1 ± 0.5	< 0.001
Total Cholesterol (mg/dL)	199 ± 39	199 ± 39	198 ± 40	200 ± 36	0.949
Fasting Triglyceride (mg/dL)	138 ± 83	128 ± 75	149 ± 102	138 ± 48	0.081
HDL (mg/dL)	53 ± 15	55 ± 16	51 ± 15	52 ± 12	0.048
LDL (mg/dL)	125 ± 37	125 ± 38	126 ± 39	124 ± 30	0.883
VFA^a^ (cm^2^)	130 ± 39	129 ± 41	131 ± 39	129 ± 34	0.963
WC (cm)	94.5 ± 13	94.1 ± 13	94.9 ± 13	94.7 ± 12	0.909
Thai DM risk score (points)	9 ± 4	8 ± 4	9 ± 3	10 ± 3	0.003
Thai CV risk score^b^ (points)	4 (1–10)	3 (1–7)	4 (1–9)	10 (2–19)	< 0.001

^a^ Available in 268/512 overall subjects (107/220 in NGT, 112/191 in IGT, 49/101 in DM)

^b^ Displayed as median (IQR)

### Diagnostic accuracy of HbA1c

**Fig. 3:**
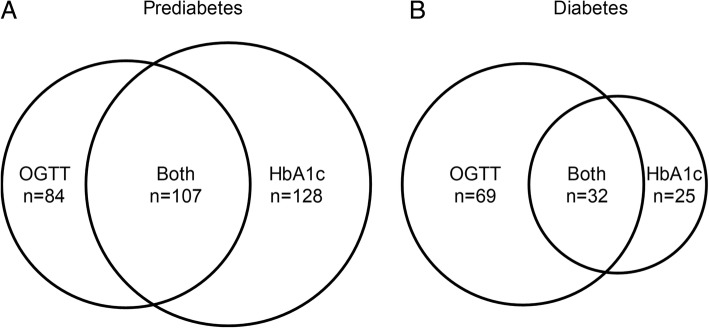
Diagram showing discordants in diagnosis of **a**) Prediabetes defined by 2-h OGTT and HbA1c of ≥6.5%. **b** Diabetes defined by IGT and HbA1c of 5.7–6.4%

Figure 3 shows diabetes prevalence was twice as high when defined by OGTT as when defined by HbA1c (19.7% versus 11.1%, *p*-value < 0.001). However in diagnosing prediabetes, HbA1c as diagnostic criteria for prediabetes (HbA1c 5.7–6.4%) can detect up to 235 (45.9%) subjects while only 191 (37.3%) subjects were classified as IGT from OGTT. Sensitivity, specificity, positive predictive value, and negative predictive value for detecting selected type 2 diabetes mellitus at different HbA1c thresholds are displayed in Table 2. The sensitivity of recommended cut-off HbA1c (HbA1c ≥ 6.5%) is only 32% (95% CI 23–41%) but with high specificity of 94% (95% CI 92–96%) by using OGTT as the reference diagnosis. The best optimal cut-off HbA1c threshold to diagnose diabetes per OGTT criteria was found at 6.2% (Youden’s index 0.391).

**Table 2:** Sensitivity, specificity, positive likelihood ratio, and negative likelihood ratio for detecting selected type 2 diabetes mellitus at different HbA1c thresholds

HbA1c (%NGSP))	Sensitivity	Specificity	Positive Likelihood Ratio	Negative Likelihood Ratio	Youden’s Index
5.7	78% (70–86%)	48% (43–53%)	1.51 (1.31–1.73)	0.45 (0.31–0.66)	0.264
5.8	74% (66–83%)	58% (53–62%)	1.75 (1.49–2.06)	0.45 (0.32–0.63)	0.319
5.9	69% (60–78%)	65% (60–69%)	1.96 (1.63–2.36)	0.47 (0.35–0.64)	0.340
6.0	63% (54–73%)	72% (67–76%)	2.25 (1.81–2.78)	0.51 (0.39–0.66)	0.351
6.1	57% (48–67%)	79% (75–83%)	2.68 (2.09–3.44)	0.54 (0.43–0.68)	0.360
6.2	54% (45–64%)	85% (81–88%)	3.55 (2.66–4.74)	0.54 (0.43–0.67)	0.391
6.3	46% (36–55%)	89% (86–92%)	4.07 (2.80–5.75)	0.61 (0.51–0.74)	0.344
6.4	38% (28–47%)	91% (89–94%)	4.42 (2.95–6.62)	0.68 (0.58–0.80)	0.291
6.5	32% (23–41%)	94% (92–96%)	5.21 (3.24–8.38)	0.73 (0.64–0.83)	0.256

### The agreement between HbA1c and OGTT

**Fig. 4:**
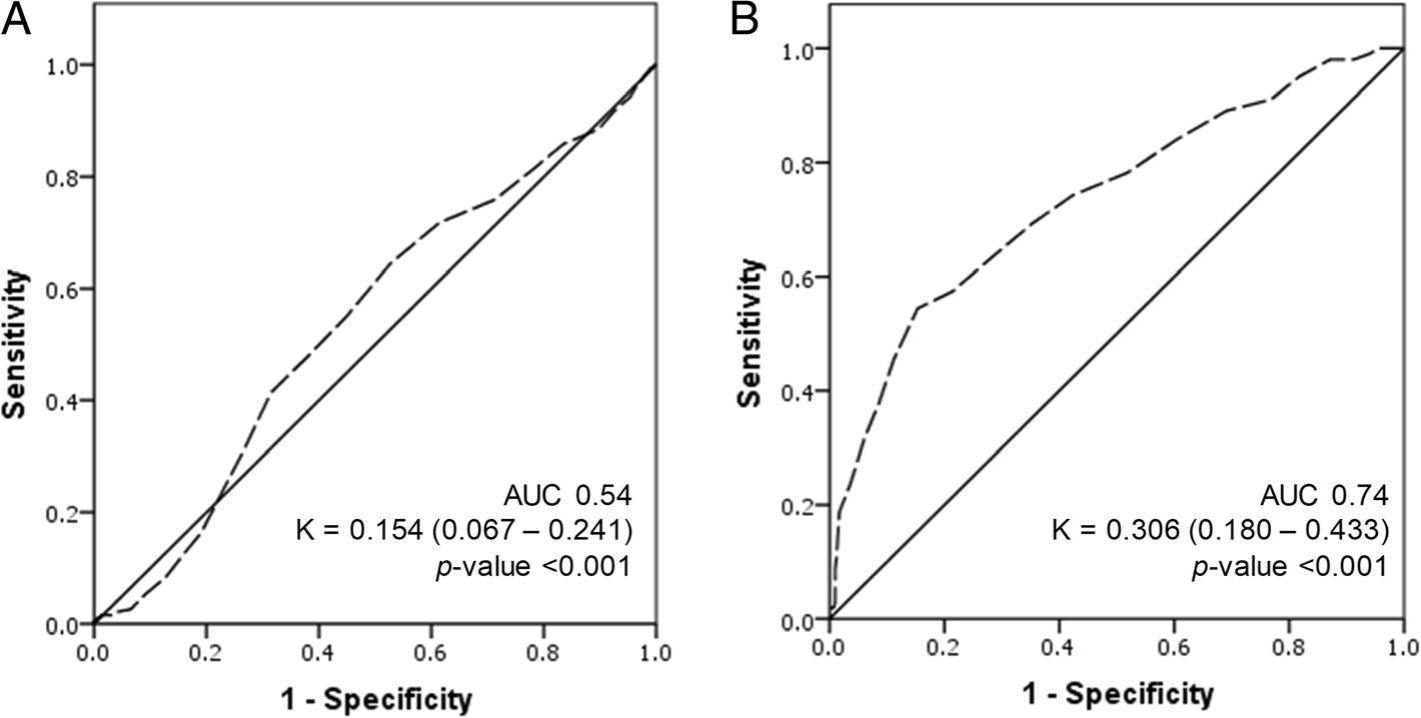
Area under the receiver operating characteristic curve (AUC) for **a**) concordance between OGTT and HbA1c in diagnosis of prediabetes (AUC = 0.54) **b**) concordance between OGTT and HbA1c in diagnosis of diabetes (AUC = 0.74)

We evaluated the agreement represented by the ROC curves between the classification of prediabetes and diabetes defined by OGTT and HbA1c in Fig. 4. The diagnostic ability of HbA1c for prediabetes and diabetes are represented by the ROC curves. In diabetes group, the AUC was 0.74 indicating that HbA1c was an acceptable test to diagnose diabetes. The agreement, represented by kappa value of 0.306 (95% CI 0.180–0.433), was considered a fair agreement between the two tests. However in diagnosing prediabetes, AUC from the ROC curve is 0.54. Thus, HbA1c could not be used to discriminate subjects with IGT. The Kappa value of 0.154 (95% CI 0.067–0.241), indicated that there was no agreement between the two tests in diagnosing prediabetes.

## Discussions

In this study we found dysglycemia in almost 60% of high-risk adult Thai patients based on the results of OGTT in a hospital setting. Compared to OGTT, HbA1c has lower sensitivity but higher specificity in diagnosing diabetes. The optimal cut-off HbA1c point to diagnose diabetes was found at 6.2% per OGTT criteria which is lower than the current HbA1c-based criteria of diabetes. Such findings suggest that physicians should advocate OGTT as a screening tool for the identification of dysglycemic status in high-risk Thai patients. Alternatively, a lower cut-off point for HbA1c might be suitable for high-risk Thai patients.

The natural history of T2DM is characterized by a progressive decline in beta-cell function, a process that is accelerated by obesity. In vivo studies in humans indicated that there is a 70% decrease in beta-cell glucose responsiveness by the time that individual has developed IGT [19]. Primary prevention by lifestyle modifications and pharmacological therapy had been shown to be effective, especially in IGT patients [20–22]. Early detection for primary prevention is therefore critical to prevent future diabetes and cardiovascular diseases. The low reproducibility, intense sweetness problem and inconvenience in terms of costs and time consumption often hampered physicians’ decision to routinely use OGTT. However, previous studies from our group and others confirmed the possibility and practicality to use this test as an essential diagnostic procedure for both diagnosis and screening of diabetes [14, 23–25]. Currently, IGT does not receive much attention from healthcare providers [26]. Perceived barriers for both patients and clinicians should be corrected and the individuals with IGT should be identified to receive interventions on modifiable risk factors for T2DM and cardiovascular disease.

Glycated hemoglobin was endorsed as one of the criteria for diagnosis of prediabetes and diabetes by ADA in 2010 [27] and by the World Health Organization (WHO) in 2011 [8] based on its equal sensitivity and specificity to other methods as a predictor of prevalent retinopathy. However, it needed to emphasize that the quality assurance tests are in place and assays are standardized to criteria aligned to the international reference values [28]. Also, it should be ensured that there are no conditions present which preclude accuracy of HbA1c measurement [29]. The use of HbA1c can avoid the requirements for individual to fast or to have adequate carbohydrate intakes before OGTT testing. Hemoglobinopathies especially thalassemia which affects 5–10% of individuals from Southeast Asia is known to interfere with some HbA1c assay [30]. But the prevalence of major thalassemia (beta-thalassemia and beta-thalassemia associated with other Hb anomalies) varies among different regions in each country [31]. Therefore, HbA1c is still a valuable tool for early diagnosis of dysglycemia in the Southeast Asia region if we understand the limitations of its use.

Our results in terms of discordance between HbA1c and OGTT were consistent with other studies in Asian population. A study in Japan showed concordance of HbA1c to diagnose prediabetes with Kappa value of 0.10 and AUC of 0.65 [32]. In Singapore, a study showed that HbA1c is more consistent in prediabetes but also had lower sensitivity [33]. Those findings indicated that a substantial number of diabetes cases would be missed by using the HbA1c test alone compared with OGTT [34, 35]. It should be noted that the agreement in diagnosing prediabetes and diabetes do not reflect how one test would be better than the other. A previous study in Chinese population suggested the use of both HbA1c and plasma glucose [36]. Some studies recommended raising HbA1c cut-off points for prediabetes and diabetes in obese subjects [37, 38]. We recommend using both HbA1c and OGTT to capture the full spectrum of dysglycemic status in high-risk patients. A recent population-based study in Vietnam [39] showed the prevalence of diabetes to be at 12.3% and prediabetes at 40.1% based on HbA1c criteria which are similar to our study. Therefore, prediabetes detected by the HbA1c should be further explored in the Asia Pacific region in order to maximize the role of HbA1c in early detection of dysglycemic status. With respect to HbA1c criteria for diabetes, our study suggests additional research on the optimal HbA1c cut-off points to identify diabetes in high-risk Southeast Asia patients.

Various DM risk scores based on clinical characteristics have been developed to identify individuals at high risk of having undiagnosed T2DM. However, risk scores developed in Caucasian populations might not apply to populations of other ethnic groups. A validated DM risk score in Thai population [15] was developed from a cohort study of employees of a state enterprise, the EGAT in which male made up more than 75% of total participants [16]. The included variables were age, sex, BMI, waist circumference, history of hypertension, and history of diabetes in parents or siblings. Our findings in this study confirmed the clinical utility of this simple risk score for different populations which were at higher risk of developing T2DM and with the majority being female. Around one fourths of subjects with very-high Thai diabetes risk score of ≥11 were found to have diabetes from OGTT. Therefore, screening patients using the simple score can be effective in identifying subjects at risk. However, our data also suggested that at-risk individuals identified in hospital settings should receive OGTT screening even those with low to moderate risk classifications (scores less than 6 out of 17).

The authors acknowledge some limitations in the present study. Firstly, the inherit weakness of poor repeatability from OGTT itself and single testing episode only might impact the misclassification of glycemic status. However, our results reflect real-life practice which limited the chance to repeat OGTT in daily service. Secondly, our study assumed that a single OGTT represents the gold standard for the presence of the disease is debatable. The decision of which test to use for diagnosis of prediabetes and diabetes is left to clinical judgement of physicians because each diagnostic test has advantages and disadvantages. Thirdly, this study is based on high-risks individuals in outpatients setting only. Thus, the prevalence in this study would differ from that of the general healthy population. Fourthly, there is also emerging evidence that mid-OGTT glucose time points may be equally or even more predictive of future risk of diabetes or retinopathy than the 2-h glucose level [40, 41]. Our present study evaluated data from the traditional post-load 2-h plasma glucose level. The role and cut-off for mid-OGTT glucose level need to be further studied in other populations. Finally, the data are retrospective cross-sectional in nature and we cannot conclude the overall progression of future diabetes, cardiovascular diseases, or other future co-morbidities for each individual. Further prospective follow up study should be conducted to evaluate our approach in using both OGTT and HbA1c to capture dysglycemia in high-risk patients and evaluate risks for long-term diabetic and cardiovascular complications.

## Conclusions

Our study found that diabetes prevalence is almost 2 times higher when diagnosed using OGTT than when using HbA1c which implies the limitations of HbA1c as a screening tool for diabetes in high-risk Thai patients. This is the first study to explore the role of HbA1c in diagnosing dysglycemic status in high-risk Thai patients. OGTT should continue to advocate as a screening tool for identification of dysglycemic status in particular population.

## Abbreviations

ADA: American Diabetes Association
AUC: Area under curves
BMI: Body mass index
CVD: Cardiovascular disease
DETECT-2 study: Detection strategies for type 2 diabetes and impaired glucose tolerance study
EGAT: Electricity Generating Authority of Thailand
FPG: Fasting plasma glucose
HbA1c: Glycated hemoglobin
IFG: Impaired fasting glucose
IGT: Impaired glucose tolerance
NGSP: National glycohemoglobin standardization program
NGT: Normal glucose tolerance
OGTT: Oral glucose tolerance test
ROC curve: Receiver operating characteristic curve
T2DM: Type 2 diabetes
VFA: Visceral fat area
WC: Waist circumference
WHO: World Health Organization
